# Modes of Death in Punctured Wounds of the Heart

**Published:** 1898-09-17

**Authors:** 


### MODES OF DEATH IN PUNCTURED WOUNDS OF THE HEART.

It is, we think, a matter of some interest to consider the various modes of death in cases of punctured wound of the heart. First of all, however, it must be remembered that death may not take place at all, and that even when a wound is ultimately followed by fatal consequences the issue may be delayed a very considerable time. Again, it must be noted that, although death may be caused by sudden arrest of the action of the heart, this does not occur as a result of direct injury to its muscular structure, but rather from shock to its nervous mechanism. The two main causes of death in stabs of the heart are internal hæmorrhage, and the interference with the action of the heart which results from the filling of the pericardium with blood. Hæmorrhage in such cases is mostly internal, and takes place into the cavity of the pleura; the same injury which punctures the heart also opening the pleura, into the cavity of which the blood is poured in such volume that fatal syncope may occur almost immediately, without pain, and may be without any sensation at all beyond the dreamy state that accompanies fading consciousness. The other great mode of death in punctured wound of the heart is interference with its action, in consequence of the accumulation of blood within the pericardium. In regard to this it is to be noted that the blood which does the mischief in such cases does not always come from the cavity of the heart. Quite a small wound may cause death, even one which does not penetrate the cavity of the heart, if it wound one of the large arteries which meander over its surface, for when one of these is opened blood may be driven into the pericardium with the full force of the ventricle, and may exercise such pressure on the outer surface of the heart as to prevent the auricles filling again, and thus the circulation may be brought to a standstill, even though the muscular structure of the heart may flutter on for a considerable time. When death takes place in this way the symptons may not be so much those of syncope as of suffocation, the respiration being gradually interfered with, even while the circulation in the brain remains sufficient for consciousness to be retained.

